# Current Strategies to Improve Chimeric Antigen Receptor T (CAR-T) Cell Persistence

**DOI:** 10.7759/cureus.65291

**Published:** 2024-07-24

**Authors:** Soren K Ghorai, Ashley N Pearson

**Affiliations:** 1 N/A, Eastside Preparatory School, Kirkland, USA; 2 Biomedical Sciences, University of Michigan Medical School, Ann Arbor, USA

**Keywords:** immune evasion, car-t persistence, adaptive immunity, car-t therapy, immunotherapy

## Abstract

Chimeric antigen receptor T (CAR-T) cell therapy has transformed the field of immunology by redirecting T lymphocytes toward tumor antigens. Despite successes in attaining high remission rates as high as 90%, the performance of CAR therapy is limited by the survival of T cells. T cell persistence is crucial as it sustains immune response against malignancies, playing a critical role in cancer treatment outcomes. This review explores various approaches to improve CAR-T cell persistence, focusing on the choice between autologous and allogeneic cell sources, optimization of culture conditions for T cell subsets, metabolite adjustments to modify T cell metabolism, the use of oncolytic viruses (OVs), and advancements in CAR design. Autologous CAR-T cells generally exhibit longer persistence but are less accessible and cost-effective than their allogeneic counterparts. Optimizing culture conditions by promoting T_SCM_ and T_CM_ cell differentiation has also demonstrated increased persistence, as seen with the use of cytokine combinations like IL-7 and IL-15. Metabolic adjustments, such as using 2-deoxy-D-glucose (2-DG) and L-arginine, have enhanced the formation of memory T cells, leading to improved antitumor activity. OVs, when combined with CAR-T therapy, can amplify CAR-T cell penetration and persistence in solid tumors, although further clinical validation is needed. Advances in CAR design from second to fifth generations have progressively improved T cell activation and survival, with fifth-generation CARs demonstrating strong cytokine-mediated signaling and long-lasting persistence. Understanding the underlying mechanisms behind these strategies is essential for maximizing the potential of CAR-T therapy in treating cancer. Further research is needed to improve safety and efficacy and seamlessly integrate the discussed strategies into the manufacturing process.

## Introduction and background

Chimeric antigen receptor T (CAR-T) cell therapy has transformed the field of immunology with outstanding success in treating hematological cancers. The idea of using a patient’s T cells to treat cancer began to take shape in the early 1980s. Initial research focused on genetically modifying T cells to better recognize and target cancer cells. However, it wasn’t until 2010 that this concept advanced with the development of chimeric antigen receptors (CARs). CARs are cell-surface receptors designed to navigate T cells toward a specific antigen. The extracellular domain of the CAR originates from an antibody fragment, allowing the cell to recognize tumor-associated antigens (TAAs). Tumors with low or no major histocompatibility complex (MHC) expression can be challenging for T cells to attack. However, CAR-T cells target cancer cells independently of MHC presentation and bind directly to TAAs. Once T cells are harvested from the donor’s blood, the CAR construct is introduced into T cells through viral vector transduction. The engineered CAR-T cells are then cultured and expanded ex vivo with supplementation of cytokines before being injected into the patient. Since the first FDA-approved CAR therapy, Kymriah, was approved in 2017, many similar therapeutics, such as Yescarta, Tecartus, and Breyanzi, have been introduced. These treatments have had remarkable success, with patients achieving complete remission (CR) 30-70% of the time and, in some trials, over 90% [1]. However, many studies still depict CARs as ineffective because of a lack of persistence. 

The clinical successes of CAR-T therapy have been linked to the ability of T cells to proliferate and survive within the patient’s body. Favorable outcomes depend on the persistence of CAR-T cells after initial infusion, meaning poor persistence will limit their efficacy [2]. A phase I study administered CAR-T therapy to 14 patients suffering from metastatic ovarian cancer, yet no tumor reduction was observed. Correspondingly, the transferred T cells did not persist much beyond five days, confirming that the lack of persistence significantly reduced their antitumor activity [3]. Another study providing CAR-T therapy to six patients with metastatic neuroblastoma saw minimal tumor response due to limited T cell persistence [4]. Both of these studies highlighted inadequate T cell persistence as a crucial issue to be addressed in future studies. Sustained persistence ensures that T cells remain active to mount an effective immune response, thus providing long-term protection against malignancies. The longer CAR-T cells can expand and remain cytotoxic, the stronger the anti-tumor response is and the higher the likelihood of achieving remission and patient survival. Considering that cancer is a long-term challenge, the duration of the immune response is even more important. Thus, this review paper will explore strategies to increase persistence.

## Review

This article was previously posted to the chemrXiv preprint server on May 13, 2024.

Autologous or allogeneic: choosing a cell source

**Figure 1:**
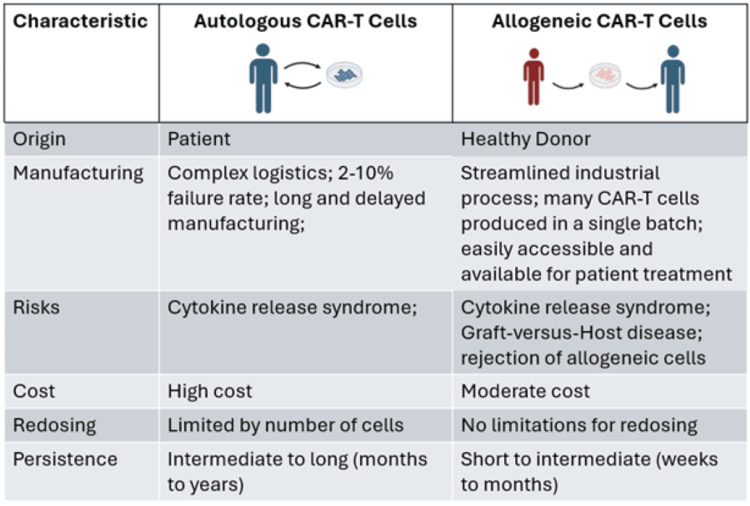
Comparison between autologous vs allogeneic CAR-T cell Autologous cells have longer manufacturing processes, higher costs, and limited redosing but exhibit stronger persistence and a lower risk for CRS. Allogeneic cells are a cheaper, more accessible patient treatment option but are less personalized and display shorter persistence, with a higher risk for CRS and GvHD. CRS, cytokine release syndrome; GvHD, graft-versus-host disease; CAR-T, chimeric antigen receptor T

Choosing a source of CAR-T cells is important to maximize persistence. The source and components of T cells influence the properties of CAR-T cells, so they should be carefully selected. CAR-T cells are classified as autologous (derived from the host) or allogeneic (derived from a donor); both of these types of cells have certain advantages and limitations that have been observed in clinical trials (Figure 1). 

On a long-term basis, autologous T cells outperform allogeneic-sourced cells, showing better durability in treatment outcomes. The CALM trial analyzed the effects of allogeneic CAR-T cells on 25 patients with B-cell acute lymphoblastic leukemia (B-ALL) over four years, observing an overall response rate of 48% [5]. Conversely, the ELIANA clinical trial studied autologous CAR-T cells in 75 patients with B-ALL, finding a high remission rate of 81% [6]. Thus, autoCAR-T cells may provide some benefit in treatment over extended periods of time. A secondary advantage of autologous cells is the diminished risk of immune rejection [7]. Donor T cells carry the potential risk of life-threatening graft-versus-host disease (GvHD) [8]. This is because allogeneic T cells may recognize the recipient’s tissues as foreign due to human leukocyte antigen (HLA) disparities, triggering an immune response against the host cells. A clinical study observed that 21.4% of patients experiencing relapsed/refractory acute lymphoblastic leukemia (ALL) who underwent alloCAR-T therapy experienced acute GvHD [9]. Additionally, externally sourced T cells may be rapidly destroyed by the patient’s immune system, decreasing persistence [8,10]. Hence, autologous CAR-T cell therapy generally corresponds with longer persistence due to the absence of an allogeneic reaction [8]. Yet autologous therapies require a tailored manufacturing process with well-known disadvantages. Autologous CAR-T cell manufacturing has 2-10% failure rates resulting from contamination, operator error, and equipment failure in commercial settings [10]. Autologous cell collection also has a high cost and long manufacturing processes, delaying the availability of treatment [8]. As a result, accelerated cell manufacturing strategies are being employed to decrease assembly mistakes, duration, and cost. Production facilities are automating their manufacturing process to reduce human error and failure rates and offer fresh, non-cryopreserved products for patients [11]. Additionally, companies use high-speed processes to minimize the long manufacturing times. For example, CAR-T cells cultured with the “T-Charge” platform save production time by shortening the expansion period ex vivo [11]. CAR-T cells made with the T-Charge platform still perform exceptionally well, exhibiting 98% response rates for patients with relapsed/refractory multiple myeloma and 71% persistence rates beyond 12 months [11]. Regardless, the manufacturing process for autologous T cells remains an important issue to address. Meanwhile, allogeneic CAR-T cells offer certain advantages due to decreased cost and scale manufacturing ability. Allogeneic therapies allow for many cells to be harvested from the donor that is then cryopreserved, making treatments readily available for use and decreasing the time to production [8]. The systematic production of allogeneic T cells is expected to reduce cost and expand accessibility for CAR-T cell therapy [8]. Autologous T cell collection can only be used to treat the donor patient, whereas allogeneic T cell manufacturing creates multiple product batches for many patients [8]. Allogeneic T cells can be injected again if the initial infusion is insufficient, which is especially crucial for patients whose disease recurs, necessitating a second dose. T cells taken from a patient who has undergone prior medical treatment may be limited in numbers, thus affecting initial administration doses and any reinfusion that may be necessary [7].

Clinical trials demonstrate that autologous T cells persist longer than allogeneic products in treating B-ALL. With allogeneic CAR-T cells, only 14% of patients had detectable CAR-T cells after 42 days; only one patient exhibited persistence beyond 120 days [10]. In contrast, the median persistence of tisagenlecleucel (autologous CAR-T cell medication) was 168 days, with some CAR-T cells persisting longer than 20 months [10]. Other in vivo trials of relapsed/refractory ALL in humans demonstrated the presence of cytokine release syndrome (CRS) after CAR-T cell treatment. CRS is indicated by high amounts of inflammatory cytokines such as interleukin-6 (IL-6) and interferon-γ (IFN-γ) and can become life-threatening [9]. In a retrospective study, many more patients experienced severe CRS (grade ≥ 3) from autoCAR treatment than alloCAR treatment [9]. The median peak IL-6 level from the autoCAR cells (161.6 pg/mL) exceeded the peak level from the alloCAR group (38.7 pg/mL) [9]. The overall remission rate was similar, with the CR rate being 88.2% for the autoCAR group and 100% CR rate for alloCAR [9]. However, a contrasting prospective study found CRS occurring in 57% of patients treated with autoCAR-T cells and 91% of those administered with alloCAR-T cells [10]. The study dosed seven children and 14 adults with UCART19, an allogeneic CAR-T therapy used to treat B-ALL [12]. Not only did 19 patients (91%) suffer from CRS, but grade 1 acute skin GvHD occurred in two patients, and grade 1 or 2 neurotoxicity occurred in eight patients (38%) [12]. Thus, more research is necessary to determine the relationship between CAR-T cell source and the risk of CRS and other adverse events. 

Various gene editing methods (TALENs, Zinc-finger nucleases, and CRISPR/Cas9) have been used with allogeneic transplants to reduce the risk for GvHD and enhance T cell persistence [13]. Due to the manufacturing drawbacks of autoCAR-T cells, allogeneic cells should continue to be developed as a cheaper alternative. Gene editing of allogeneic T cells should be explored, especially to minimize life-threatening risks like GvHD and short persistence, which could lead to relapse. 

Optimizing culture medium for T cells in vitro

CAR-T cell persistence is heavily dependent on T cell differentiation, of which there are many T cell subsets. Before encountering their cognate antigen, immature T cells are referred to as naïve T (T_N_) cells. After an antigen is encountered, T_n_ cells activate and differentiate into stem cell memory T (T_SCM_) cells, central memory T (T_CM_) cells, effector memory T (T_EM_) cells, or eventually terminal effector T (T_EFF_) cells, each with varying implications for CAR-T efficacy [1]. Recent work concluded that T_SCM_ and T_CM_ cells have longer persistence and stronger anti-tumor activity than T_EM_ and T_EFF_ counterparts [14]. Specifically, one clinical trial saw CD19-CAR-modified CD8+ T_SCM_ cells exhibit improved fitness and sustained antitumor responses against systemic ALL [14]. As a result, procedures for expanding T cells have been optimized to promote more T_SCM_ and T_CM_ cells. This has been accomplished through the strategies explored below. 

Before T cell activation, T_n_ cells mainly use oxidative phosphorylation (OXPHOS) and fatty acid oxidation (FAO) in mitochondria [15]. Cell metabolism is optimized for minimal energy consumption for cells in resting states like T_N_ cells. T_N_ cells use imported nutrients to produce energy through OXPHOS, which breaks down nutrients like fatty acids, amino acids, and glucose [16]. Upon encountering an antigen, T_n_ cells activate and transform into T_EFF_ cells, triggering an accelerated metabolism and promoting aerobic glycolysis [17]. However, memory cell subsets remain similar to T_N_ cells in that they largely depend on OXPHOS as opposed to glycolysis [18]. Recent studies indicate that the sustained elevation of glycolysis hinders the development of long-lived memory cells [19]. Pushing T cells toward an irreversibly differentiated state causes an inability to persist post-adoptive cell transfer (ACT) [20]. Therefore, strategies should shift the metabolic processes from glycolysis toward OXPHOS to achieve sustained persistence and induce more T_SCM_ and T_EM_ cells [21].

Metabolite adjustments

Multiple studies have explored metabolite adjustments to restrict or interfere with glycolysis and improve the ratio of T_SCM_ and T_CM_ cells. 2-Deoxy-D-glucose (2-DG) suppresses glycolysis by replacing the 2-hydroxyl group with hydrogen. Utilizing 2-DG during T cell activation to limit the reliance on glycolysis pushed CD8+ T cells toward a differentiation pathway, favoring the formation of T_SCM_ and T_CM_ memory cells [22]. The inhibition process preserves the formation of long-lived memory CD8+ T cells, helping to improve the efficacy and survival of CAR-T cells. Similarly, intracellular L-arginine concentrations enhance the differentiation of T_SCM_ and T_CM_ cells with longer persistence and stronger anti-tumor activity in murine models. One study used L-arginine supplementation in the cell medium to promote a switch from glycolysis to OXPHOS in CAR-T cells. The metabolic shift toward OXPHOS contributed to a higher proportion of memory cells with increased survival ability and stronger antitumor cytotoxicity [23]. Moreover, adding 6-diazo-5-oxo-L-norleucine (DON) to the cell culture enhances OXPHOS and reduces glycolytic activity [24]. This resulted in more T_CM_ subsets and stronger anti-tumor activity in vivo [24]. Furthermore, recent studies indicate that using PhysiologixTM xeno-free (XF) hGFC (Phx), a human growth factor, facilitates T cell proliferation and CAR-T cell expansion [25]. CAR-T cells were compared to cells conditioned in Phx or HS to determine a difference in anti-tumor response in a murine xenograft model [25]. T cells cultured in Phx had improved persistence in vivo and superior cytotoxic capabilities in vitro [25]. Specifically, the dipeptide carnosine found in Phx augments the persistence of T cells and shifts them toward an oxidative state, thus enriching the T_CM_ cell subset.

The common characteristic behind these studies is the metabolic increase of OXPHOS and potential inhibition of glycolysis. This improves T cell differentiation toward T_SCM_ and T_CM_ cells for superior persistence and antitumor potential. In clinical practice, one study reported that T_SCM_ enriching protocol significantly expanded CAR-T_SCM_ cells in patients with B-ALL, resulting in prolonged anti-tumor responses for long-term remissions [26]. A similar investigation emphasized memory T cell subsets as a potential biomarker for CAR-T cell clinical efficacy. After patients with chronic lymphocytic leukemia were administered with CAR therapy, transcriptomic analyses revealed that stem cell memory T cells (T_SCM_ and T_CM_) proportions were notably higher in patients with complete or partial response (PR) than in patients with no response [27]. Self-renewing properties of memory T cells contributed to extreme longevity, as memory-induced infused CAR-T cells persisted in the peripheral blood of CR patients beyond five years [27]. The discussed clinical data demonstrates how T_SCM_ and T_CM_ cells enhance CAR-T cell persistence, yielding superior antitumor responses and long-term remissions. 

Cytokine optimization

Additionally, various cytokine combinations have been tested to manipulate metabolic changes and improve the ratio of T_SCM_ and T_CM_ cells during the CAR-T cell expansion phase. IL-2 has been considered the “gold standard” growth factor, though it triggers a shift from OXPHOS to glycolysis [1]. Other cytokine combinations have additionally improved persistence in vivo by promoting OXPHOS and blocking glycolysis. Using IL-7 and IL-15 in clinical studies led to higher proportions of T_SCM_ and T_CM_ than just IL-2, which saw more significant amounts of T_CM_ and T_EM_ [20]. A separate study confirmed this, where IL-7 facilitated the highest levels of the T_SCM_ subset compared to other pro-inflammatory cytokines [21]. A trial in vivo demonstrated that IL-12 modulates the expression of CD62L on activated T cells, resulting in superior persistence and proliferation compared to IL-2 [20]. Additionally, in vivo administration of IL-15 and IL-21-treated T cells exhibited phenomenal persistence and the most effective tumor elimination in vivo. However, it is interesting to note that IL-2 improved the accumulation of CAR-T cells in vitro [21], even though IL-2-treated CAR-T cells display reduced anti-tumor cytotoxicity. Further experiments are required to determine whether other cytokine conditions can outperform the standard IL-2-manufactured CAR-T. Understanding the mechanisms underlying the promotion of memory cells is equally important. Aquaporin-9 (APQ9) is crucial for CD8+ T cell proliferation and survival, as it sustains ATP levels within T cells to enhance OXPHOS [28]. IL-7 has been shown to induce the expression of APQ9 in CD8+ T cells to sustain their growth through OXPHOS and improve their cell proportions [28]. Similarly, IL-15 is proposed to upregulate the glycogen synthase kinase-3β/Wnt pathway to enhance memory T cell maturation and proliferation [29]. IL-12 and IL-21 facilitate memory T cell development by activating the STAT4 and STAT3 pathways, respectively, enhancing mitochondrial biogenesis to stimulate OXPHOS-induced cell differentiation [30,31]. 

Oncolytic viruses 

Viral-based gene therapy strategies have attracted scientific and clinical attention. Oncolytic viruses (OVs) mediate anti-tumor effects through direct tumor cell lysis, causing the secretion of tumor-associated antigens and IFNs, which contribute to antitumor immunity [32]. As standalone treatments, OVs have yielded only modest anti-tumor effects in patients. This is largely due to the restricted entry of OVs into tumors, their limited persistence within the host, and the patient’s antiviral response [33]. However, OVs can be used in combination to enhance the efficacy of other therapies, including CAR-T cell therapy, to induce a stronger anti-tumor immune response [33]. The immunosuppressive tumor microenvironment (TME) is characterized by physical barriers, such as IL-10, which limit CAR-T cell infiltration and diminish their cytotoxic function [34]. However, OVs can stimulate immune activity in the TME to increase the presence and proliferation of T cells at the tumor site [35]. By inducing immune activation, OVs facilitate infiltration into solid tumors and enhance their susceptibility to systemic treatment, supporting the combination of immunotherapies for optimal antitumor activity. In this way, OVs help to remodel the TME, making it more conducive to T cell activity and improving CAR-T cell function. Specifically, OVs can be genetically modified to express pro-inflammatory cytokines to modulate adaptive immunity, exhibit immune co-stimulators to improve CAR-T cell activation, and present immune checkpoint inhibitors to alleviate T cell suppression [36,37]. This helps activate APCs within the TME, including dendritic cells and macrophages, which are primed by tumor-associated antigens released from cells after OV-induced oncolysis [38,39]. Additionally, the release of pro-inflammatory IFN-γ from viral infection upregulates MHC expression to improve CAR-T cell activation [40]. 

Preclinical studies show that integrating CAR-T cells with OVs directs attention to the tumor-specific expressed tumor antigen limitations that arise from the use of CAR alone. One trial paired CAR-T cells directed toward CA IX and an oncolytic adenovirus delivering chemokine ligand 5 (CCL5) and IL-12 to investigate anti-tumor potential [33]. Results demonstrated that the combined therapy, Ad5-ZD55-hCCL5-hIL12, caused a modest suppression of human-derived tumors in murine models. Ad5-ZD55-hCCL5-hIL-12 and CA9-CAR-T cells enhanced CAR-T cell penetration within solid tumors, restrained cancer growth, and extended overall persistence [33]. Hence, this study demonstrates effective results in integrating OAV with CAR-T cells for anti-tumor treatment. Another study examined CAR-T cells in conjunction with OVs equipped with CCL5 and the proinflammatory IL-15 [41]. Researchers observed that the administration of the OAV accelerated cell death in neuroblastoma tumors treated by CAR-T therapy [41]. The adenovirus caused CCL5 and IL-15 to be secreted, which activated CAR-T cells and increased their persistence, thus lengthening overall mouse survival [41]. A different study tested the treatment of OAV with chemokine CXCL11 against glioblastoma models using immunodeficient mice [42]. The combination of therapies elicited robust antitumor effects and increased infiltration of CD8+ T lymphocytes [42]. 

While these preclinical studies have shown favorable results, further investigation should assess the potency of OVs with CAR-T therapy in human immune systems. An ongoing study (NCT03740256) was designed to analyze the safety concerns of using OVs in humans. The trial combines HER2-targeted CAR-T cells and an oncolytic adenovirus, CAdVEC, to administer treatment for patients with HER2-positive cancer. This trial is still in the recruitment phase, although its results may provide insight into treating patients with a combination of CAR-T and OVs.

CAR design

**Figure 2:**
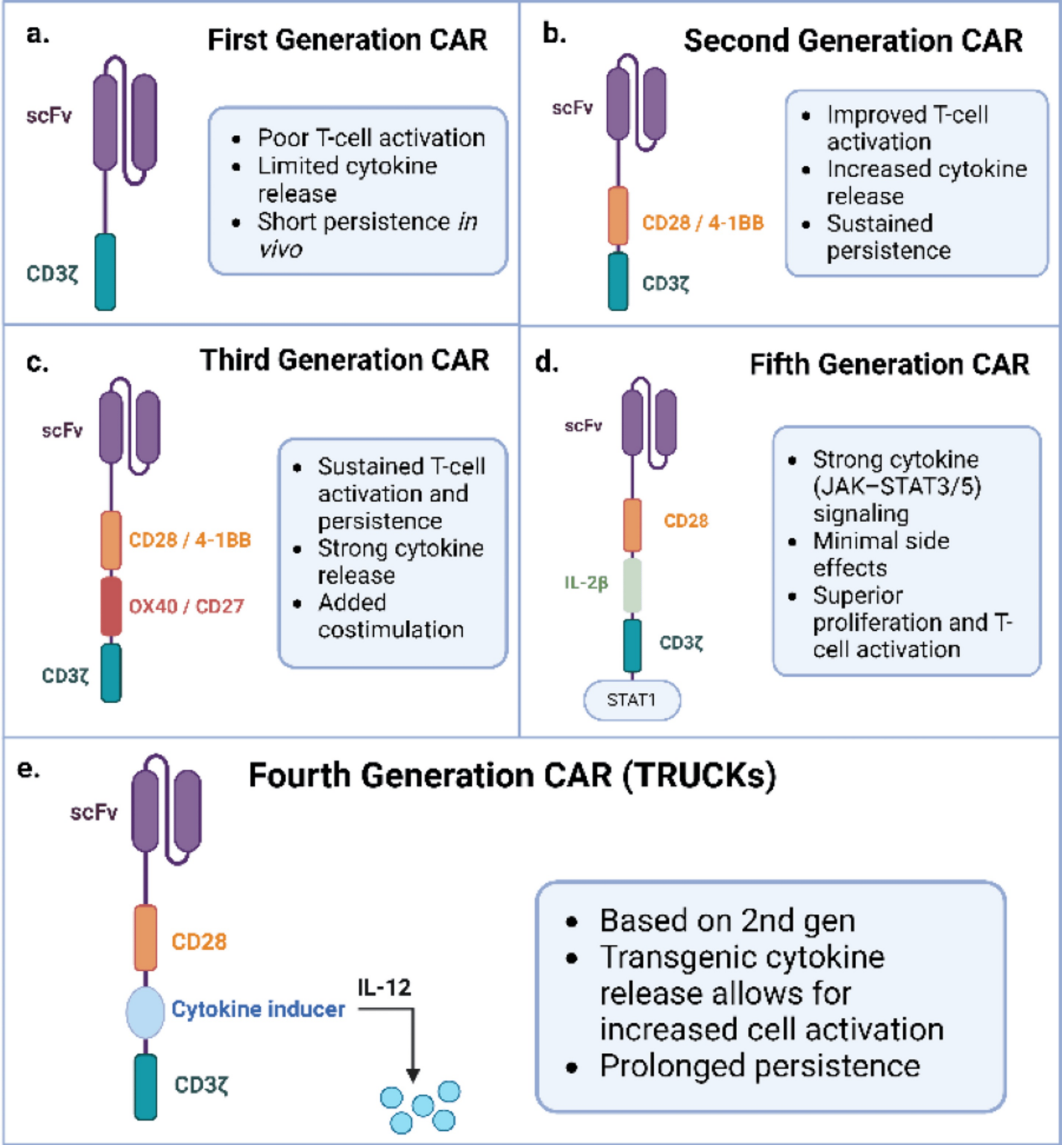
CAR-T construct designs (A) First-generation CARs contained an scFv and an additional signaling domain CD3ζ. (B) The second generation included a secondary costimulator, CD28, or tumor necrosis factor ligand superfamily member 9 (4-1BB). (C) Third-generation CARs were composed of two secondary costimulatory domains. (D) Fourth-generation TRUCKs were modeled after second-generation CARs and contained gene expression cassette-inducing cytokines. (E) The fifth generation of CARs consisted of an IL-2 receptor β-chain domain and a binding location for Janus kinase-signal transducer and activator of transcription pathway (JAK-STAT3/5) [16]. CAR-T, chimeric antigen receptor T; scFv, single-chain variable fragment; CD3ζ, cluster of differentiation 3ζ; CD28, cluster of differentiation 28; TRUCK, T cells redirected for universal cytokine-mediated killing; IL, interleukin

Since the creation of CARs in 1993, the survival of engineered T cells has posed a serious obstacle to successful clinical outcomes. As a result, several generations of CARs have been introduced, each with its own design and attributes that prolong cell survival [43]. The first generation was composed of a single-chain variable fragment (scFv) and a cytoplasmic CD3ζ signaling domain (Figure 2A) [44]. However, it was unsuccessful in activating the T cells through the CD3ζ domain. First-generation CARs cannot produce a sustained anti-tumor response or cytokine release due to poor signaling [45].

To strengthen signal transduction, the second generation of CARs included a costimulatory domain, such as CD28 or 4-1BB, to promote T cell activation, survival, and proliferation of the engineered cells (Figure 2B) [46]. Kymriah and Yescarta, both FDA-approved therapies for B-ALL and large B-cell lymphoma (LBCL), are second-generation CAR-T medications. Second-generation CARs demonstrate the importance of including a costimulatory domain to achieve stronger persistence and overall function of CAR-T cells. A clinical trial administered CAR-T cells containing 4-1BBζ to 14 patients with chronic lymphocytic leukemia, finding that the cells persisted for over four years among patients who achieved CR [2]. Another use involving the 4-1BB costimulatory receptor promoted the development of the T_CM_ subset with enhanced metabolic efficiency through FAO and OXPHOS pathways [47]. Additionally, autologous T cells expressing a CD28ζ domain yielded T_SCM_ and T_CM_ cells with increased glycolytic metabolism. Overall, CARs incorporating 4-1BBζ and CD28ζ tend to promote the differentiation of T_SCM_ and T_CM_ cells, respectively, leading to enhanced persistence. The important idea from the second generation is that including a costimulatory domain is crucial for substantive T cell activation and improvement of anti-tumor response. 

Third-generation CARs improved costimulation by including two co-stimulatory signaling domains (Figure 2C) [16]. Incorporating the OX40, ICOS, or CD27 as secondary costimulatory domains increases the expansion and persistence of resting T cells through proinflammatory pathways [48]. In murine models, third-generation CARs mount a more robust antitumor response and contribute to longer-surviving mice [49]. Specifically, ICOS signaling improves CAR-T cell persistence in solid tumors compared to a single costimulatory domain by increasing cytokine release, such as IFN-γ and IL-22 [16]. A clinical study demonstrated third-generation CARs to perform moderately well, with two patients achieving CR and one patient arriving at a PR [50]. The study observed T cell persistence of up to 12 months with slightly improved antitumor activity, suggesting a benefit from the additional costimulatory domain [51]. Hence, the preclinical and clinical outcomes of third-generation CARs remain promising. 

The fourth iteration of CARs, termed T cells redirected for universal cytokine-mediated killing “TRUCKs,” was modeled after second-generation CARs. TRUCKs feature a gene expression cassette that can be activated to produce a transgenic cytokine (Figure 2D) [16]. Cytokines, including IL-12, are delivered directly to the tumor to improve immune response [51]. TRUCKs can minimize systemic side effects commonly associated with widespread cytokine release, such as CRS, by producing cytokines near the tumor. After the initial CAR-T cell response, tumor relapse can occur if CAR-T cells fail to detect cancer cells with minimal receptor expression [51]. As a result, secretion of inducible IL-12 from TRUCKs brings immune cells toward the microenvironment to eradicate the remaining parts of the tumor [51]. Stimulating cytokines alters the TME, leads to prolonged activation of CARs, and shields overactive T cells from programmed cell death, thus increasing persistence [52]. Patient outcomes were examined in a clinical study in which 16 patients with metastatic melanoma were administered with low doses of TRUCKs [53]. Ten out of 16 patients (63%) displayed an objective response, with nine patients exhibiting a PR and one patient achieving CR [53]. These clinical results are robust and indicate the improvement of the direct secretion of IL-12. Thus, TRUCKs offer comparable positive patient treatments to third-generation CARs but achieve this through localized cytokine delivery rather than a supplementary costimulatory domain. However, in terms of side effects, third-generation CARs are associated with higher risks for CRS due to preferential secretion of IL-6, which is a disadvantage compared to using TRUCKs [54]. 

Finally, fifth-generation CARs, or “next-generation,” include an IL-2 receptor β-chain domain and a binding location for STAT3 (Figure 2E) [16]. This structure contributes to strong cytokine (JAK-STAT3/5) signaling within the specific tumor sites while minimizing systemic side effects [53]. IL-2 signaling influences two important aspects of immune response: T cell differentiation and memory recall responses [55]. Fifth-generation CAR modifications contribute to improved T cell activation, persistence, and proliferation through cytokine-mediated signaling pathways [52]. One recent clinical trial (ANTLER) undergoing phase 1 treated relapsed/refractory B cell non-Hodgkin lymphoma patients with “next-generation” CAR-T therapy, reporting an overall response rate of 92% (≥4 HLA matches) and a complete response rate of 46% (≥4 HLA matches) (NCT04637763). The study also observed promising safety results, as no instances of grade 3 or higher CRS were reported, and zero patients contracted GvHD. Thus, “next-generation” CAR-T therapy offers the most positive patient outcomes in comparison to TRUCKs, third-generation, and previous CAR constructs. Additionally, the strong patient tolerance and lack of side effects from fifth-generation CARs suggest a favorable advancement in CAR-T therapy. 

**Figure 3:**
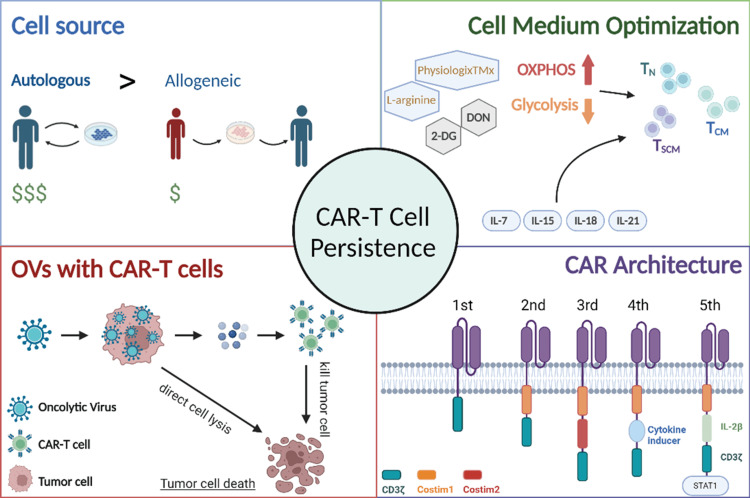
Approaches to increase CAR-T cell persistence Upper left: choosing between autologous or allogeneic cells as a source for CARs. Upper right: optimizing cell medium to increase T cell differentiation toward memory cells. Bottom left: oncolytic viruses overcome immunosuppressive mechanisms within the microtumor environment. Bottom right: generations of CAR-T cell construct designs. CAR-T, chimeric antigen receptor T

Persistence can be improved through multiple strategies, such as choosing an appropriate source of T cells, optimizing the cell medium with cytokine and metabolite adjustments, using OVs, and developing CAR architecture (Figure 3). These strategies have demonstrated significant promise, and their safety and efficacy should continue to be examined.

Procedures

Literature Search Strategy

A comprehensive literature search was conducted to identify relevant studies on strategies to improve CAR-T cell persistence. Due to the heterogeneity of clinical studies, a narrative review was chosen instead of a meta-analysis. Large variability among CAR-T cell modifications, costimulatory domains, and treatment protocols makes it difficult to standardize data for quantitative synthesis. On the other hand, many studies provide context-specific findings that are not flexible enough for numerical analysis. A narrative review was chosen to examine strategies in T cell persistence, highlight study-specific limitations, and emphasize overall challenges in implementing the discussed strategies. Multiple academic databases were used, including PubMed, Google Scholar, and ScienceDirect. PubMed and ScienceDirect provided an extensive collection of biomedical literature and peer-reviewed research. Google Scholar captured a broader range of publications, including theses and conference papers that may not be indexed in traditional databases. The search was performed using a combination of keywords and phrases such as “CAR-T cell therapy,” “T cell persistence,” “immunoediting of CAR-T cells,” and “CAR-T cell metabolic adaptations.” Keywords were used in various combinations to refine search results. The time frame for the search for clinical studies was set from 2005 to 2024 to ensure that both early foundational studies and the latest advancements were included. Studies were referenced if they met the following criteria: (1) focused on strategies to enhance CAR-T cell persistence, (2) involved experimental or clinical data, and (3) provided quantitative or qualitative assessments of CAR-T cell persistence. Studies involving human subjects or relevant animal models were eligible, provided they offered relevant insights into CAR-T cell persistence and patient outcome. However, human trials were prioritized. Studies that reported challenges with CAR-T therapy were also incorporated to provide a balanced perspective on the literature. The discussed strategies were chosen based on their potential impact on CAR-T therapy and previous measurable improvements in T cell persistence. Strategies were also selected if they addressed significant challenges in existing CAR-T cell research. The outcomes of interest in evaluating studies were focused on CAR-T cell persistence in vivo, antitumor activity, patient response rates (progressive disease (PD), stable disease (SD), PR, complete response (CR)), survival rates, and toxic side effects (e.g., CRS and GvHD). These outcomes help assess the effectiveness of each strategy aimed at increasing CAR-T cell persistence and optimizing therapeutic results. 

## Conclusions

CAR-T therapy has become recognized as a revolutionary immuno-therapeutic tool that targets a wide variety of cancers. However, obstacles like a lack of T cell persistence must be overcome to achieve superior clinical results. Cancer is a long-term challenge characterized by its ability to evolve and resist treatment over time. Given these circumstances, the sustained activity of T cells becomes important to attain long-lasting immune responses for CR of disease. Our knowledge of each approach and its underlying mechanisms is crucial for leveraging these strategies to improve the persistence of CAR-T cells. Changing things like the cell culture medium or adding metabolite adjustments are challenging to incorporate on a large scale. These changes must be carefully evaluated to ensure they do not compromise the quality or safety of the treatment. Media formulations that support T cell growth without expensive additives should be prioritized, and manufacturers should switch to more affordable cell culture media that can reduce widespread costs. To increase scalability, automated systems for monitoring metabolite levels can help maintain consistency without manual intervention, which could result in human error and exacerbate production failures. Development of autologous T cells is already expensive, although allogeneic T cell production remains a favorable solution. Also, a phased implementation approach can help make these upgrades more cost-effective, with incremental improvements instead of incurring all expenses at once. Additionally, focusing on efficiency improvements can lead to significant cost savings. By identifying and eliminating production bottlenecks, improving cell culture workflows, and incorporating automation, overall production efficiency can be enhanced to lower operational costs.

This review discussed strategies to extend CAR-T cell persistence. Approaches such as selecting an appropriate source of cells, enriching culture conditions, using combinatorial treatments, and optimizing CAR architecture have made significant progress in improving T cell persistence. Future research should focus on refining these strategies to implement them in the CAR manufacturing process.
